# Dynamics of the Physicochemical Characteristics, Microbiota, and Metabolic Functions of Soybean Meal and Corn Mixed Substrates during Two-Stage Solid-State Fermentation

**DOI:** 10.1128/mSystems.00501-19

**Published:** 2020-02-11

**Authors:** Cheng Wang, Changyou Shi, Weifa Su, Mingliang Jin, Bocheng Xu, Lihong Hao, Yu Zhang, Zeqing Lu, Fengqin Wang, Yizhen Wang, Huahua Du

**Affiliations:** a National Engineering Laboratory of Biological Feed Safety and Pollution Prevention and Control, Key Laboratory of Animal Nutrition and Feed, Ministry of Agriculture, Key Laboratory of Animal Nutrition and Feed Science of Zhejiang Province, Institute of Feed Science, College of Animal Science, Zhejiang University, Hangzhou Zhejiang, People’s Republic of China; USDA-Agricultural Research Service, Boyce Thompson Institute, Cornell University

**Keywords:** microbiota, metabolic functions, nutritional value, two-stage solid-state fermentation

## Abstract

Substantial annual economic loss in livestock production is caused by antinutritional factors in soybean meal and corn mixed substrates, which can be degraded by microbial fermentation. Although considerable efforts have been made to explain the effects of fermentation on soybean meal and corn-based feed, the dynamics of the physicochemical characteristics, microbiota, and metabolic functions of soybean meal and corn mixed substrates during solid-state fermentation remain unclear. Here, multiple physicochemical analyses combined with high-throughput sequencing were performed to reveal the dynamic changes that occur during a novel two-stage solid-state fermentation process. Generally, inoculated bacteria rapidly proliferated in the initial 12-h aerobic fermentation (*P = *0.002). Notably, most nutritional changes occurred during 12 to 24 h compared to 0 to 12 h. Second-stage anaerobic fermentation increased the bacterial abundance and lactic acid content (*P < *0.00). Bacillus spp., Enterococcus spp., and Pseudomonas spp. were predominantly involved in the maturation of the fermented mixed substrates (*P < *0.05). Additionally, the available phosphorus exhibited the greatest interaction with the microbial community structure. Cellular processes and environmental information processing might be the main metabolic processes of the microbiota during this fermentation. An *in vivo* model further evaluated the growth-promoting effects of the fermented products. These results characterized the dynamic changes that occur during two-stage solid-state fermentation and provided potential references for additional interventions to further improve the effectiveness and efficiency of solid-state fermentation of feed.

**IMPORTANCE** Solid-state fermentation (SSF) plays pivotal roles not only in human food but also farm animal diets. Soybean meal (SBM) and corn account for approximately 70% of the global feed consumption. However, the nutritional value of conventional SBM and corn mixed substrates (MS) is limited by antinutritional factors, causing substantial economic loss in livestock production. Although emerging studies have reported that SSF can improve the nutritional value of SBM-based substrates, the dynamic changes in the physicochemical features, microbiota, and metabolic functions of MS during SSF remain poorly understood, limiting further investigation. To provide insights into the dynamics of the physicochemical characteristics and the complex microbiome during the two-stage SSF of MS, multiple physicochemical analyses combined with high-throughput sequencing were applied here. These novel insights shed light on the complex changes that occur in the nutrition and microbiome during two-stage SSF of MS and are of great value for industrial feed-based practices and metabolomic research on SSF ecosystems.

## Abstract (precis)

Solid-state fermentation (SSF) plays pivotal roles not only in human food but also farm animal diets. Soybean meal (SBM) and corn account for approximately 70% of the global feed consumption. However, the nutritional value of conventional SBM and corn mixed substrates (MS) is limited by antinutritional factors, causing substantial economic loss in livestock production. Although emerging studies have reported that SSF can improve the nutritional value of SBM-based substrates, the dynamic changes in the physicochemical features, microbiota, and metabolic functions of MS during SSF remain poorly understood, limiting further investigation. To provide insights into the dynamics of the physicochemical characteristics and the complex microbiome during the two-stage SSF of MS, multiple physicochemical analyses combined with high-throughput sequencing were applied here. These novel insights shed light on the complex changes that occur in the nutrition and microbiome during two-stage SSF of MS and are of great value for industrial feed-based practices and metabolomic research on SSF ecosystems.

## INTRODUCTION

Soybean meal (SBM) and corn account for approximately 70% of the global feed consumption. Approximately 772.37 million tons of soybean meal and corn were used in global livestock production in 2018 (Alltech, Nicholasville, KY). However, the utility of conventional SBM and corn mixed substrates (MS) is limited by several antinutritional factors (ANFs), which inhibit the bioavailability of nutrients and reduce animal health status (1). In 2018, it was estimated that $32.9 billion was lost due to the adverse effects of ANFs on farm animals (Alltech, Nicholasville, KY). Soybean antigenic proteins, mainly glycinin and beta-conglycinin, have been shown to cause antigenic protein hypersensitivity, which is attributed to gut injury, inflammation, and diarrhea (2). Trypsin inhibitors (TIs) hinder the activity of the endogenous enzyme trypsin, thereby causing digestive disorders (3). By binding positively charged molecules, phytic acid can reduce nutrient digestibility (4). Additionally, some carbohydrate-based ANFs, such as cellulose and amylose, can interfere with nutrient digestibility and increase nutrient emission (5). Overall, ANFs inhibit the bioavailability of nutrients and cause physiological disorders.

Solid-state fermentation (SSF), an age-old biotechnology, has been widely used to promote the nutritional quality of feed by promoting nutrient utilization and decreasing ANF levels (6). In terms of feed quality improvement, SSF remains much more economical and beneficial than other methods. The global feed production by SSF approached 2 million tons in 2018 (National Engineering Research Center of Biological Feed). The nutritional quality of the fermented feed depends on the type of microorganism used for inoculation. Aspergillus spp. and Bacillus subtilis are commonly used in ANF degradation due to their capacity to produce enzymes such as protease, amylase, xylanase, pectinase, and amylase under aerobic conditions (7). Some research has demonstrated the degradation of ANFs and hydrolysis of macronutrient factors by microbes in SBM-based substrates (8, 9). Additionally, lactic acid (LA) bacteria are commonly used in fermented feed to produce organic acids, especially LA, under anaerobic conditions (10). However, few studies have combined the features of both aerobic and anaerobic SSF to optimize the quality of fermented feed. In addition, the dynamic changes in physicochemical characteristics, microbial community structure, and metabolism of MS during SSF remain unclear, impeding further research.

Therefore, SBM and corn were used as the main fermented substrates, which were inoculated with an effective combination of B. subtilis and Enterococcus faecium to achieve a novel two-stage SSF process (first stage, aerobic fermentation; second stage, anaerobic fermentation). Multiple physicochemical analysis methods combined with high-throughput sequencing were applied to provide insights into the dynamic changes in physicochemical features and complex microbiomes during the two-stage SSF of MS. Furthermore, weaned piglets were used as an *in vivo* model to investigate the effects of fermented MS (FMS) on growth performance, nutrient utilization, and anti-inflammatory activity. These findings provide insights into the potential changes in the physicochemical characteristics, main bacteria, and metabolism of FMS during two-stage SSF and provide valuable information for industrial feed-related practices and microbiome research on SSF.

## RESULTS

### Fermentation inoculum selection and process design.

Our previous study rationally screened B. subtilis CW4 from different fermented foods (11). To further improve the rate of ANF degradation, the effects of microbes alone or in combination with a neutral protease on 24-h protein degradation were compared (see Fig. S1A in the supplemental material). The results showed that inoculation with 10^7^ CFU/g Saccharomyces cerevisiae, 10^7^ CFU/g E. faecium CWEF, and 300 U/g neutral protease was not effective enough for protein degradation. Furthermore, different LA bacteria were added for the following 48 h for anaerobic fermentation to increase the levels of probiotic and microbial metabolites (Fig. S1B). Interestingly, the inclusion of 10^7^ CFU/g E. faecium and 10^7^ CFU/g Lactobacillus plantarum for two-stage fermentation did not hinder the degradation effects of CW4. Additionally, E. faecium was more effective at reducing pH than was *L. plantarum.* Therefore, a novel and effective inoculum combination and fermentation process (10^7^ CFU/g B. subtilis CW4, aerobic fermentation for 24 h; and 10^7^ CFU/g E. faecium CWEF, anaerobic fermentation for 48 h) was selected for further study.

10.1128/mSystems.00501-19.1 FIG S1 Process design and microbial metabolite analysis (*n* = 4). (A and B) SDS-PAGE of FMS conducted by different inoculations (A) and processes (B). (C to G) Levels of microorganisms, pH (C), lactic acid (D), and enzymes (E to G) during two-stage SSF. The dotted line represents the time of addition of E. faecium. (A) Lane 1, mixed substrates; lane 2, CW4 for 24 h; lane 3, neutral protease for 24 h; lane 4, S. cerevisiae for 24 h; lane 5, S. cerevisiae plus neutral protease for 24 h; lane 6, ZJU 13 for 24 h; lane 7, CWEF plus neutral protease for 24 h. (B) Lane 1, mixed substrates; lane 2, CW4 for 24 h; lane 3, CWEF plus *L. plantarum* for 72 h; lane 4, CW4 for 24 h plus *L. plantarum* for 48 h; lane 5, CWEF plus *L. casei* for 72 h; lane 6, CW4 for 24 h plus CWEF for 48 h; lane 7, CW4 for 24 h plus *L. casei* for 48 h. Download FIG S1, TIF file, 2.6 MB. Copyright © 2020 Wang et al. 2020 Wang et al. This content is distributed under the terms of the Creative Commons Attribution 4.0 International license.

### Chemical composition.

SBM and corn are widely consumed by farm animals and contain different C/N ratios. To obtain novel substrates that contain a better C/N ratio for bacterial growth, rice dried distillers grains with solubles (rDDGS) was chosen as a supplementary ingredient. SBM, corn, and rDDGS were mixed at a ratio of 2:2:1 (Table S1). The determined nutrient content of FMS at 0 h, 12 h, 24 h, and 48 h is presented in Table 1. First-stage fermentation dynamically and markedly influenced the properties of proteins in MS. The hydrolysis rates of glycinin and beta-conglycinin were 67.50% and 74.98%, respectively, after B. subtilis treatment, while little degradation was observed during the second stage of anaerobic fermentation. In the present study, the enzyme activity of neutral protease was notably improved from 12.32 ± 2.61 (SD) to 219.42 ± 8.76 U/g during B. subtilis fermentation (Fig. S1E). The initial TI percentage was 6.12% ± 1.27%. After 24 h of fermentation, the TI concentration decreased to 0.51% ± 0.11% and further decreased to 0.24% ± 0.05% during second-stage fermentation. The neutral detergent fiber (NDF) content decreased significantly from 13.61% ± 0.64% to 9.38% ± 0.36% during the processes. Correspondingly, the phytate phosphorus content decreased from 0.37% ± 0.02% to 0.23% ± 0.03%. The total starch and amylose levels were significantly reduced by 18.37% and 54.68%, respectively.

**TABLE 1:** Analyzed nutrient content of FMS at 0, 12, 24, and 48 h

Component	Mean ± SD at time (h)^*a*^:			
	0	12	24	48
C/N ratio	10.76 ± 0.20 A	10.66 ± 0.15 AB	10.14 ± 0.15 B	9.60 ± 0.17 C
pH	6.46 ± 0.06 B	6.53 ± 0.08 B	6.76 ± 0.06 A	5.20 ± 0.13 C
Dry matter content (%)	92.47 ± 0.07	92.42 ± 0.10	92.09 ± 0.04	91.44 ± 0.09
Crude protein content (%)	28.37 ± 0.86 C	29.51 ± 0.40 BC	30.83 ± 0.69 AB	31.37 ± 0.43 A
TCA-SP content (%)	1.58 ± 0.19 D	2.89 ± 0.13 C	6.55 ± 0.18 B	10.95 ± 0.05 A
Free amino acid content (%)	0.28 ± 0.08 B	0.29 ± 0.02 B	0.77 ± 0.03 A	1.04 ± 0.21 A
Small peptide content (%)	1.26 ± 0.15 D	2.60 ± 0.19 C	5.70 ± 0.23 B	9.90 ± 0.16 A
NDF content (%)	13.61 ± 0.64 A	13.19 ± 0.13 A	9.47 ± 0.50 B	9.38 ± 0.36 B
ADF content (%)	6.24 ± 0.30	6.09 ± 0.13	5.93 ± 0.48	5.98 ± 0.39
Total starch content (%)	37.88 ± 0.85 A	36.85 ± 0.53 A	36.97 ± 0.69 A	30.92 ± 0.72 B
Amylose content (%)	4.70 ± 0.26 A	4.45 ± 0.22 A	3.46 ± 0.19 B	2.13 ± 0.17 C
Ash content (%)	6.93 ± 0.48	7.18 ± 0.73	7.62 ± 0.79	7.34 ± 0.76
Ca content (%)	0.19 ± 0.02	0.20 ± 0.04	0.18 ± 0.01	0.17 ± 0.01
Total P content (%)	0.48 ± 0.02	0.49 ± 0.02	0.51 ± 0.01	0.49 ± 0.03
Phytate phosphorus content (%)	0.37 ± 0.02 A	0.33 ± 0.03 AB	0.30 ± 0.01 B	0.23 ± 0.03 C
Available phosphorus (%)	0.11 ± 0.01 D	0.16 ± 0.01 C	0.21 ± 0.00 B	0.25 ± 0.01 A
Glycinin content (%)	70.83 ± 5.91 A	50.30 ± 3.23 B	26.15 ± 2.53 C	22.65 ± 4.18 C
β-Conglycinin content (%)	65.31 ± 4.38 A	58.09 ± 4.76 A	28.63 ± 4.65 B	16.34 ± 3.32 C
Trypsin inhibitor content (%)	6.12 ± 1.27 A	3.49 ± 0.62 B	0.51 ± 0.11 C	0.24 ± 0.05 C

a Means with different letters in each row differ at *P* < 0.05.

10.1128/mSystems.00501-19.7 TABLE S1 N and C contents and C/N ratio of fermented ingredients. Download Table S1, DOCX file, 0.1 MB. Copyright © 2020 Wang et al. 2020 Wang et al. This content is distributed under the terms of the Creative Commons Attribution 4.0 International license.

The crude protein content increased from 28.37% ± 0.86% to 31.37% ± 0.43%. Notably, the trichloroacetic acid-soluble protein (TCA-SP) concentration gradually increased from 1.58% ± 0.19% to 2.89% ± 0.13% over the 0 h- to 12-h time period. A dramatic increase was observed in the following 12 h, and the final content was 10.95% ± 0.05%. Additionally, similar trends were observed for the levels of free amino acids and small peptides (SPs), which increased by 2.71 and 6.67 times, respectively, after fermentation. In addition, the LA content and pH of the FMS reached 108.67 ± 9.39 mmol/kg and 5.21, respectively (Fig. S1D).

### Electrophoresis and microscopic observation.

**FIG 1:**
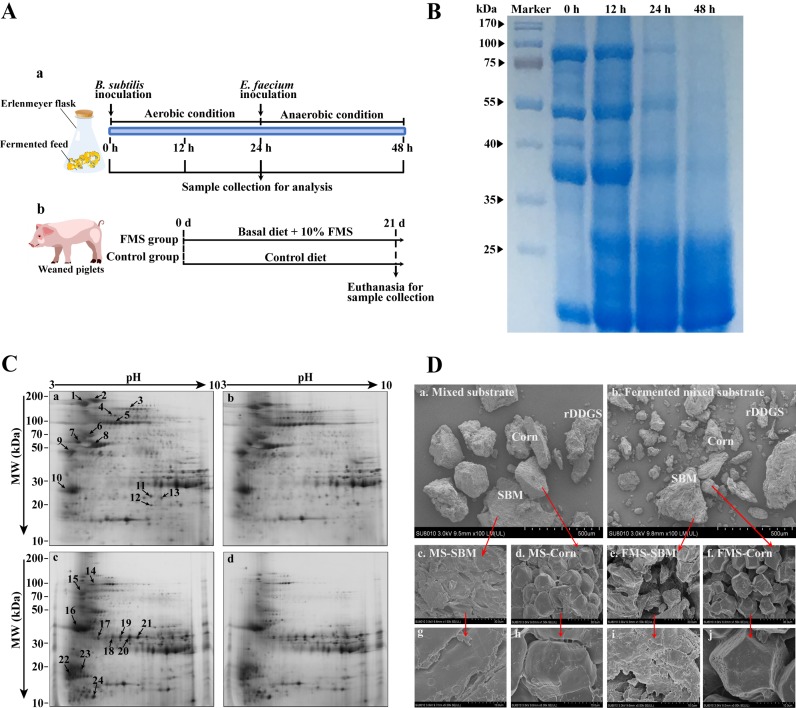
Experimental design, electrophoresis, and SEM image of FMS. (A) Experimental design. (B and C) SDS-PAGE (B) and two-dimensional (2D) electrophoretograms (C) of FMS at fermentation times of 0 h, 12 h, 24 h, and 48 h. (B) SEM images of MS and FMS after 72 h of fermentation at ×100 (a and b), ×1,500 (c to f), and ×5,000 (g to j) fold magnification. d, days; MW, molecular weight.

In the present study, the protein profile of the FMS was distributed in the range of 20 to 100 kDa (Fig. 1B). For unfermented MS, the proteins were in the range of 20 to 100 kDa. The molecular weights of the main protein fractions in the unfermented MS were approximately 80, 50, and 37 kDa. After 24 h of fermentation, the large proteins in the FMS were hydrolyzed into molecules with markedly smaller masses (<30 kDa), resulting in a decrease in the levels of large proteins (>35 kDa). The protein profile showed marginal enrichment during the anaerobic stage of fermentation compared to the aerobic stage of fermentation. Interestingly, the protein profile of FMS at 12 h was similar to that of the MS, although the chemical analysis results were different.

A total of 728 protein spots were detected in the MS sample by two-dimensional electrophoresis (2DE) (Fig. 1Ca). These spots were localized in the isoelectric point (pI) range of 3 to 10. The protein profiles changed dramatically during 12 to 24 h of MS fermentation (Fig. 1Cb and c). These results demonstrated that a considerable number of the proteins were degraded to SPs during SSF. Thirteen major proteins from the MS protein profile were selected to identify the degradation of antigenic proteins during fermentation (Fig. 1Ca) and were found to be reduced in the FMS protein profile (Fig. 1Cc). The data listed in Table 2 include the protein spot number(s), protein identity, molecular weight, pI value, MASCOT score(s), and source. Beta-conglycinin and glycinin were found in the 2DE profile of MS, and the levels of these molecules decreased in FMS. The alpha (spots 1, 14, 15, and 16), alpha′ (spot 2), and beta′ (spots 5, 19, 20 and 21) subunits of beta-conglycinin and each acidic chain and basic chain of glycinin were well resolved, detected, and identified. Spot 1 at 0 h was degraded into spots 14, 15, and 16, suggesting a decrease in the level of the alpha subunit of beta-conglycinin. Similarly, the beta subunit of beta-conglycinin (spot 5) and glycinin G4 (spot 8) decomposed into spots 19, 20, 21, and 17. Overall, the spots in Fig. 1Cc are much smaller than those in Fig. 1Ca. Upon further examination, granule-bound starch synthase 1 (spots 4), trypsin inhibitor A (spot 10), class I heat shock protein (spot 11), nucleoside diphosphate kinase 1 (spot 12), and basic 7S globulin (spot 13) were also well resolved, detected, and identified. Notably, TI A (spot 10) disappeared after 48 h.

**TABLE 2:** Spot numbers for 2D maps

Spot no.	Protein	Mol wt (kDa)	pI	MASCOT score(s)	Source
1, 14, 15, 16	α subunit of β-conglycinin	70.25	5.07	641, 310, 299, 211	Soybean
2	α′ subunit of β-conglycinin	74.28	5.47	219	Soybean
3	Sucrose-binding protein	60.48	6.42	317	Soybean
4	Granule-bound starch synthase 1	65.92	6.59	574, 686, 694	Corn
5, 19, 20, 21	β′ subunit of β-conglycinin	50.52	5.88	520, 478, 348, 461	Soybean
6	Glycinin G5	57.92	5.6	521	Soybean
7	Glycinin G3	54.36	5.46	166	Soybean
8, 17	Glycinin G4	63.55	5.29	401, 257	Soybean
9	Glycinin G2	54.36	5.46	99, 306, 364, 274	Soybean
18	Glycinin G1	55.67	5.89	340	Soybean
10, 22, 23, 24	Trypsin inhibitor A	23.99	4.99	351	Soybean
11	17.5-kDa class I heat shock protein	17.53	5.33	79	Soybean
12	Nucleoside diphosphate kinase 1	16.43	5.93	250	Soybean
13	Basic 7S globulin	46.36	8.68	172	Soybean

Scanning electron microscopy (SEM) was applied to investigate the physical structures of MS and FMS. Figure 1D shows the surface images of MS and FMS at magnification factors of ×100, ×1,500, and ×5,000. After 48 h of fermentation, more fragmental structures were detected. At the same magnification factor, SBM and corn in MS had relatively large, compact, and smooth-faced structures, while SBM and corn in FMS had smaller cracked structures and large holes.

### Changes in the bacterial community.

**FIG 2:**
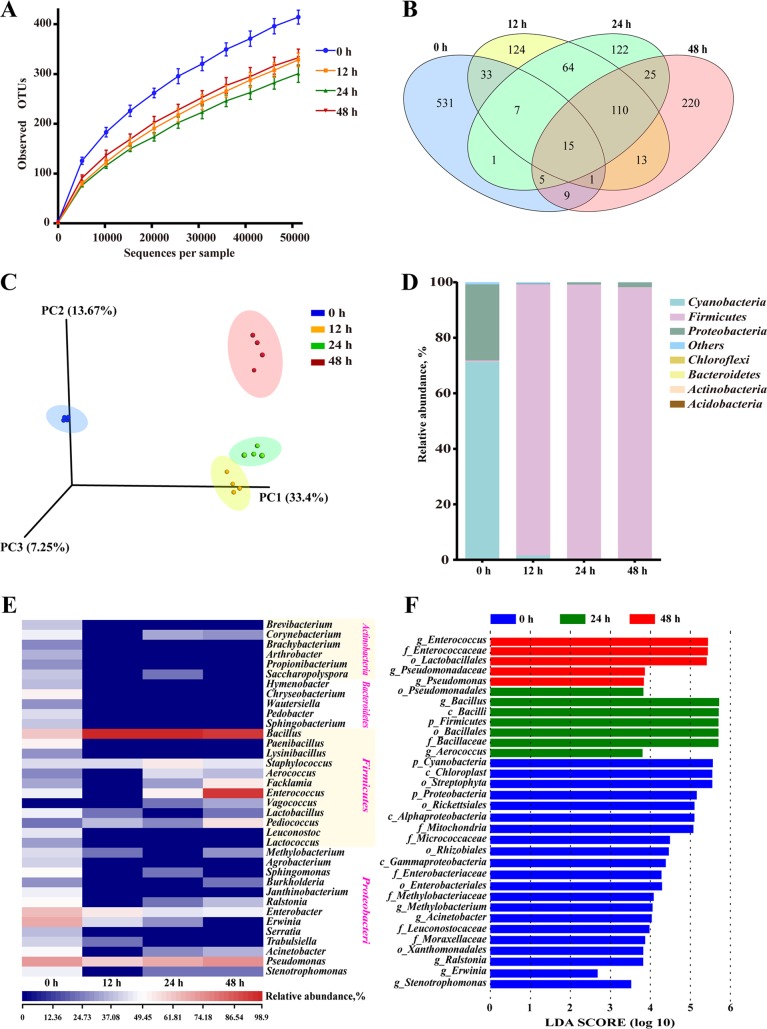
Microbial diversity and community structure during two-stage SSF (*n* = 4). (A) Observed OTU line chart. (B) Venn diagram representing the common and unique OTUs found at each fermentation time point. (C) 3D principal-component (PC) analyses of samples conducted based on unweighted UniFrac distances. (D and E) Phylum-level (D) and genus-level (E) compositions of the bacterial community in FMS. (F) LEfSe histogram showing the LDA scores (>3.5) computed for features at the OTU level. Letters indicate the taxonomy of the bacteria: *p*, phylum; *c*, class; *o*, order; *f*, family; *g*, genus.

Overall, 258,144 high-quality sequences were collated. Additionally, the general 16S rRNA operational taxonomic unit (OTU) numbers reached 1,390 based on 97% sequence similarity (Table 3). Combined with Good’s coverage index (99.6% ± 0.00%, data not shown), the results suggested that the samples exhibited abundant OTU coverage and that the sequencing depth was sufficient for analysis of the actual structure of the bacterial community during SSF. Figure 2A shows that the number of OTUs decreased after the addition of B. subtilis during initial 24 h. In contrast, the number of OTUs increased after following 24 h of anaerobic fermentation. A Venn plot (Fig. 2B) shows the common and unique OTUs in the groups. Fifteen OTUs as core genera were shared by all of the groups. The three-dimensional (3D) principal-component analysis (PCA) plot (Fig. 2C) shows that samples at 0 h, 12 h, and 48 h were well resolved and obviously distinct, while the OTUs of the samples at 12 h and 24 h exhibited fewer differences than did those of the other samples.

**TABLE 3:** Characteristics of amplicon libraries

Characteristic	Data for samples at time (h)^*a*^:				Total no.
	0	12	24	48	
No. of sequences	69,151 ± 4,299	61,811 ± 627	64,525 ± 5,021	62,657 ± 8,350	258,144
No. of OTUs	411 ± 13 A	327 ± 13 B	319 ± 16 B	333 ± 17 B	1,390
Chao1 index	676.73 ± 15.96 A	720.83 ± 57.93 A	701.71 ± 45.62 B	639.51 ± 45.99 A	
Shannon index	1.90 ± 0.21 B	0.58 ± 0.03 C	0.53 ± 0.07 C	2.42 ± 0.13 A	
Simpson index	0.50 ± 0.09 B	0.10 ± 0.01 C	0.09 ± 0.02 C	0.70 ± 0.02 A	

a Means with different letters in each row differ at *P* < 0.05.

In general, more than seven bacterial phyla were found in all the samples (Fig. 2D). In the MS samples, *Cyanobacteria* and *Proteobacteria* accounted for 99.96% ± 0.10% of the sequences. However, as fermentation progressed, *Firmicutes* rapidly became the primary members of the community, accounting for approximately 98% of the sequences.

With regard to the changes in bacterial community structure, the results at the genus level were similar to those at the phylum level (Fig. 2E). Unfermented materials contained various native bacteria, including pathogens such as Enterobacter spp. As the overall fermentation progressed, the predominant bacteria changed from *Cyanobacteria* and *Proteobacteria* to Bacillus spp., Enterococcus spp., and Pseudomonas spp. This result was consistent with the culture results for some detected microbes (Fig. S1C).

Furthermore, the linear discriminant analysis (LDA) effect size (LEfSe) results showed significantly different taxonomy among different fermentation time points (Fig. 2F). After 24 h of aerobic fermentation, the abundances of the genera *Bacillus* and Aerococcus increased significantly. After 24 h of anaerobic fermentation, *Enterococcus* spp., *Pseudomonas* spp., and *Lactobacillales* were predominant. The results of LEfSe were further verified by multiple-test correction (Fig. S2).

10.1128/mSystems.00501-19.2 FIG S2 Significant differences in microbial community (*n* = 4). (A) Bacteria at 0 h compared with those at 12 h. (B) Bacteria at 0 h compared with those at 24 h. (C) Bacteria at 0 h compared with those at 48 h. (D) Bacteria at 12 h compared with those at 48 h. (E) Bacteria at 24 h compared with those at 48 h. The extended error bar plot indicates the difference in the mean proportions of the microbial community between the two groups along with the associated confidence interval of the effect size and the *P* value of the two‐sided Welch’s *t* test (*P < *0.05). Download FIG S2, TIF file, 2.5 MB. Copyright © 2020 Wang et al. 2020 Wang et al. This content is distributed under the terms of the Creative Commons Attribution 4.0 International license.

### Bacterial metabolism of fermented mixed substrates.

**FIG 3:**
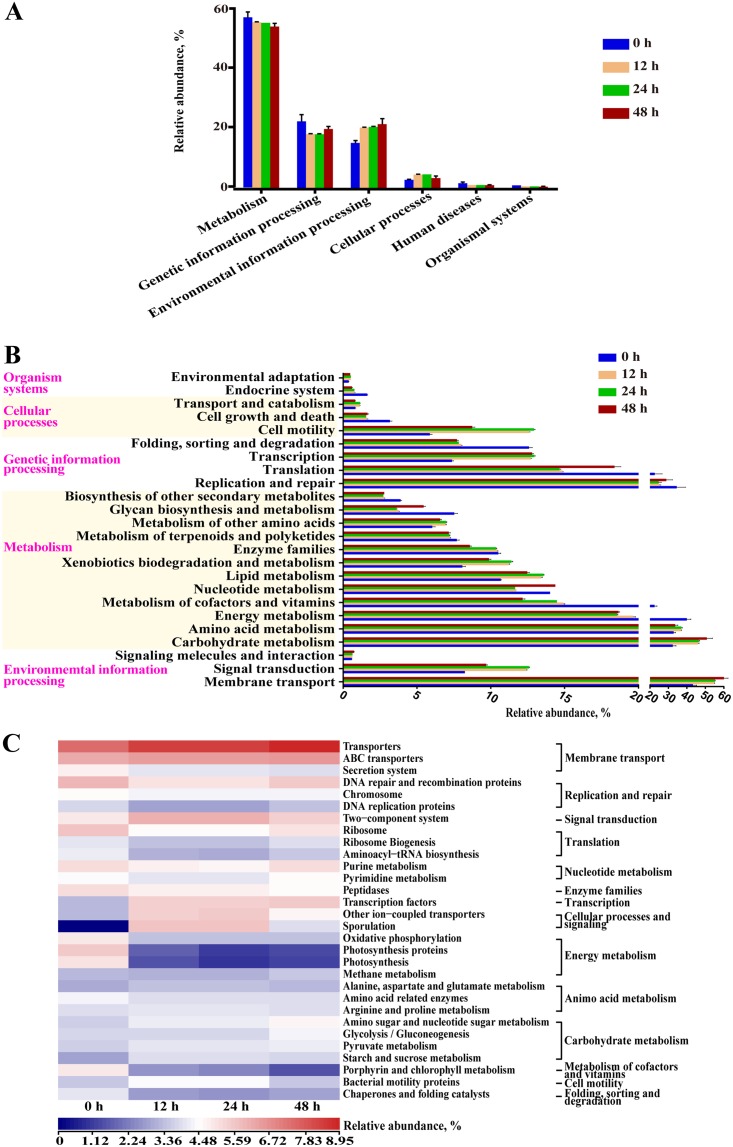
Dynamics of bacterial functional profiles during MS fermentation processes analyzed by PICRUSt (*n* = 4). (A) Level 1 metabolic pathways. (B) Level 2 KEGG ortholog functional predictions. (C) Level 3 KEGG ortholog functional predictions of the relative abundances of the top 30 metabolic functions.

The microbial metabolic functions shown in Fig. 3 were obtained based on the Kyoto Encyclopedia of Genes and Genomes (KEGG) pathway database. A majority of the predicted protein sequences ranged from 55.27% ± 1.35% to 0.72% ± 0.20% at four time points among six different metabolic functions (Fig. 3A), which represented different pathways (Fig. 3B). Notably, carbohydrate metabolism, amino acid metabolism, and membrane transport accounted for more than 30% of the enriched pathways throughout the fermentation period. Furthermore, the sequences related to amino acid metabolism, excretory system, and the transport, catabolism, and metabolism of other amino acids were significantly enriched during first-stage fermentation (*P < *0.00) (Fig. S3A). The sequences at 48 h, which were related to carbohydrate metabolism, membrane transport, and metabolism, were highly distinct from the pathways in the sample at 0 h and 24 h (*P < *0.00).

10.1128/mSystems.00501-19.3 FIG S3 Differences in gene function at the KEGG L2 (LDA, >3.0) (A) and L3 levels (LDA, >3.2) (B) (*n* = 4). Download FIG S3, TIF file, 2.9 MB. Copyright © 2020 Wang et al. 2020 Wang et al. This content is distributed under the terms of the Creative Commons Attribution 4.0 International license.

At level 3 of microbial gene functions, some differences in efficiency were observed during SSF (Fig. 3C). The abundances of a majority of the genes assigned to amino acid metabolism (alanine, aspartate, and glutamate metabolism and arginine and proline metabolism) and carbohydrate metabolism (amino sugar and nucleotide sugar metabolism, glycolysis/gluconeogenesis, pyruvate metabolism, and starch and sucrose metabolism) increased gradually during fermentation (*P < *0.05). Similarly, the genes associated with transporters, ATP-binding cassette (ABC) transporters, and transcription factors were markedly enriched from 5.13%, 3.12%, and 0.86% to 8.95%, 3.69%, and 2.37%, respectively (*P < *0.05). In contrast, the abundances of most genes related to cellular processes and signaling and energy metabolism decreased with fermentation. Interestingly, the abundances of genes involved in translation and nucleotide metabolism decreased during the aerobic fermentation period, while a considerable increase was observed following anaerobic fermentation. As expected, the gene functions related to B. subtilis (sporulation and other iron-coupled transporters) were improved after the addition of B. subtilis and reduced during second-stage fermentation. The detailed differences in gene functions are shown in Fig. S3B. The results of PICRUSt prediction at level 3 were verified by multiple-test correction (Fig. S4).

10.1128/mSystems.00501-19.4 FIG S4 Significant differences in microbial gene function at KEGG level 3 (*n* = 4). (A) Gene function at 0 h compared with that at 12 h. (B) Gene function at 0 h compared with that at 24 h. (C) Gene function at 0 h compared with that at 48 h. (D) Gene function at 12 h compared with that at 24 h. (E) Gene function at 12 h compared with that at 48 h. (F) Gene function at 24 h compared with that at 48 h. The extended error bar plot indicates the difference in the mean proportions of the microbial community between the two groups along with the associated confidence interval of the effect size and the *P* value from the two‐sided Welch’s *t* test (*P < *0.05). Download FIG S4, TIF file, 2.6 MB. Copyright © 2020 Wang et al. 2020 Wang et al. This content is distributed under the terms of the Creative Commons Attribution 4.0 International license.

### Relationship of the bacterial community with physicochemical features and metabolic functions.

**FIG 4:**
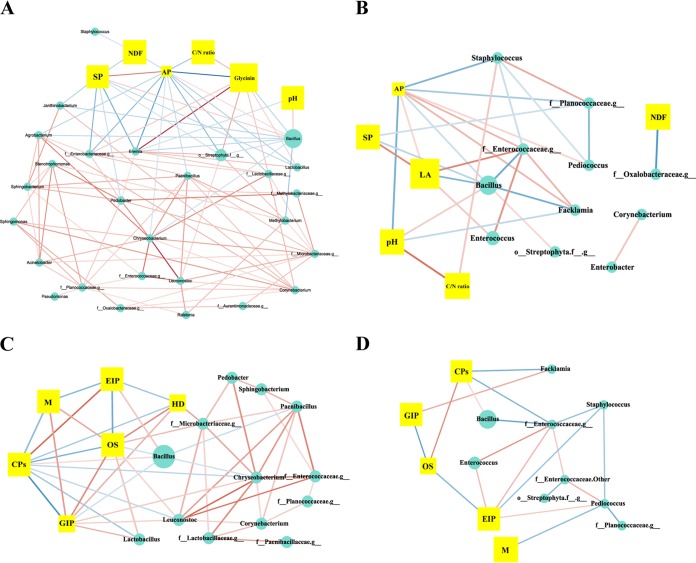
Network analysis of physicochemical characteristics, bacterial communities, and metabolic functions in two-stage SSF (*n* = 4). (A to D) The cooccurrence of physicochemical characteristics and bacterial communities under aerobic (A) and anaerobic (B) fermentation conditions and of microbial metabolism and the corresponding bacteria under aerobic (C) and anaerobic (D) fermentation. A connection indicates a significant correlation (*P* < 0.05) and Spearman’s rank (*r* > 0.8). The red lines represent positive relationships, and the green lines indicate negative relationships. The color saturation of the edges represents the correlation strength of the interaction. The node size indicates the abundances of bacteria/physicochemical characteristics/microbial metabolism. M, metabolism; CPs, cellular processes; EIP, environmental information processing; GIP, genetic information processing; SPs, small peptides; AP, available phosphorus; HD, human diseases; OS, organismal systems.

Conetwork analysis was applied to assess the relationship between physicochemical characteristics and microbial communities. The C/N ratio, available phosphorus (AP) content, SP content, pH, LA content, glycinin content, and NDF content were selected as physicochemical characteristics. During aerobic fermentation, most genera were positively related to each other (Fig. 4A). In contrast, *Bacillus* spp. exhibited a negative association with most other genera. *Bacillus* spp. were positively related to AP content, whereas most genera, such as Agrobacterium spp., Erwinia spp., Paenibacillus spp., and *Lactobacillus* spp., were negatively related to AP content. Similar patterns were found for SP and glycinin. As expected, the genera that had a negative relationship with SP and a positive relationship with glycinin were inhibited by *Bacillus* spp. Under anaerobic conditions, *Bacillus* spp. and *Enterococcus* spp. became the predominant genera (Fig. 4B). *Enterococcus* spp. had a positive relationship with LA. In contrast, *Bacillus* spp. were negatively related to SP and LA. As shown in Fig. 4C, during the 0- to 24-h period, *Bacillus* spp. were markedly positively related to environmental information processing and cellular processes. Leuconostoc spp. and *Lactobacillus* spp. were significantly enriched in the genetic information processing and organic systems, while *Bacillus* spp. were negatively associated with these pathways. Under anaerobic conditions, *Bacillus* spp. still had a positive relationship with cellular processes, while *Enterococcus* spp. were notably positively related to environmental information processing (Fig. 4D).

### Gene-level assessment.

**FIG 5:**
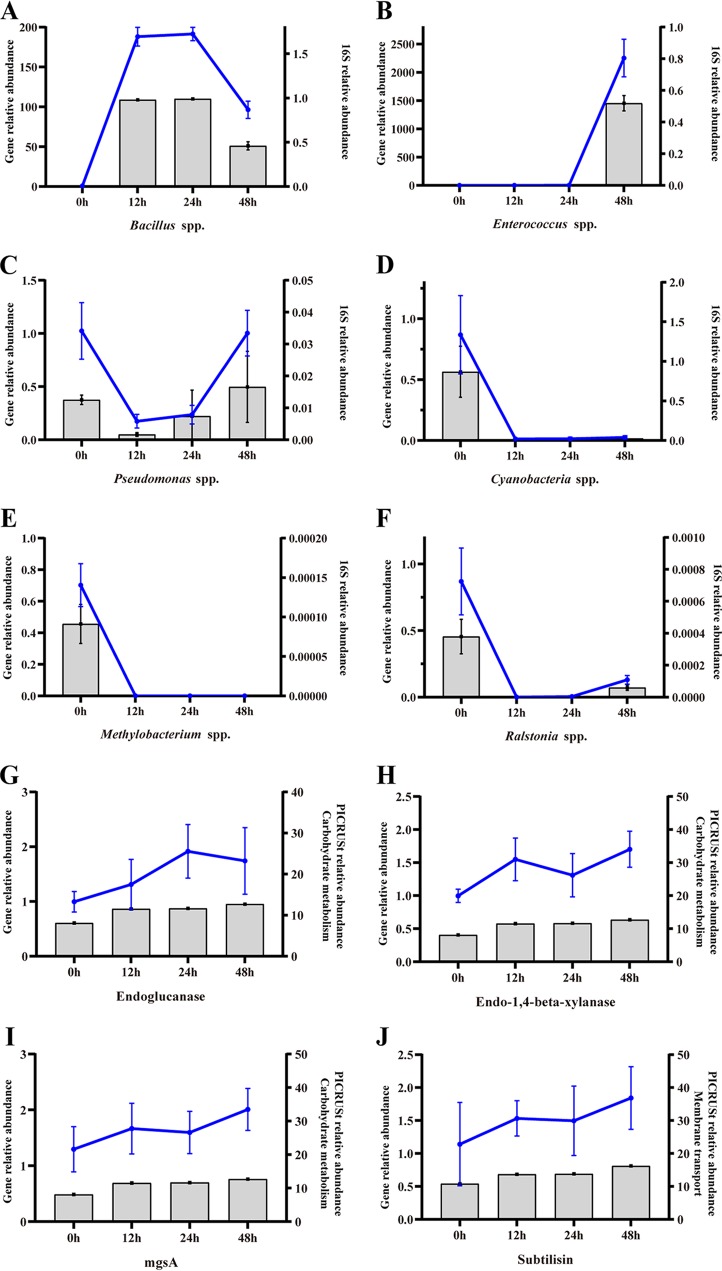
Gene-level assessment of bacteria and metabolic genes (*n* = 4). The blue line chart represents the gene level. The bar chart represents the 16S level. (A) *Bacillus* spp. (B) *Enterococcus* spp. (C) *Pseudomonas* spp. (D) *Cyanobacterium* spp. (E) *Methylobacterium* spp. (F) *Ralstonia* spp. (G) Endoglucanase. (H) Endo-1,4-xylanase. (I) *mgsA*. (J) Subtilisin.

Real-time quantitative PCR was further employed to assess the accuracy of 16S rRNA and PICRUSt analysis. The results of the gene-level assessment are provided in Fig. 5. Gene levels were tested in three core genera (*Bacillus* spp., *Enterococcus* spp., and *Pseudomonas* spp.) and three feed-native bacterial genera (Methylobacterium spp., Cyanobacteria spp., and Ralstonia spp.) during fermentation. The results of gene-level assessment were very similar to the results of the 16S rRNA analysis. In addition, some functional genes that are involved in important metabolic pathways related to fermentation were analyzed. Endoglucanase and endo-1,4-beta-xylanase contribute greatly to breaking down fiber content. MgsA is related to LA generation by LA bacteria during second-stage fermentation (12). These three genes belong to carbohydrate metabolism pathways. Subtilisin plays a critical role in protein degradation during fermentation and is an extracellular protease that is associated with membrane transport. In general, the gene-level assessment was consistent with the PICRUSt prediction.

### *In vivo* evaluation of fermented mixed substrates.

Supplementation with FMS significantly increased the average daily weight gain of piglets (Table S4). In addition, piglets fed FMS exhibited markedly lower diarrhea incidence than did the control group. In addition, higher villus height and villus height/crypt depth ratio were found in the FMS inclusion group than in the control group. Pigs in the MS group showed higher crude protein (CP) and total phosphorus (TP) digestibility than did those in the control group. Moreover, piglets fed FMS showed lower mRNA expression of the inflammatory cytokines interleukin 4 (IL-4) and IL-6 in the jejunum than did piglets in the control group (Fig. S5A). The phosphorylation of the key factors p38, IκB-α, and p65 in the jejunum was also reduced in the FMS group (Fig. S5B).

10.1128/mSystems.00501-19.5 FIG S5 Inflammation-relieving effects of FMS. Proinflammatory cytokines (A) and phosphorylation of the p38, IκB-α, and p65 proteins in the jejunum of piglets (B) (*n* = 6). Download FIG S5, TIF file, 0.7 MB. Copyright © 2020 Wang et al. 2020 Wang et al. This content is distributed under the terms of the Creative Commons Attribution 4.0 International license.

10.1128/mSystems.00501-19.10 TABLE S4 Effects of supplementation with 10% FMS on the performance of weaned piglets. Download Table S4, DOCX file, 0.1 MB. Copyright © 2020 Wang et al. 2020 Wang et al. This content is distributed under the terms of the Creative Commons Attribution 4.0 International license.

### Process design for two-stage solid-state fermentation of mixed substrates.

Figure S6 shows the process flowsheet for two-stage SSF to manufacture FMS using corn, soybean, and rDDGS as the substrates. The flowsheet is divided into three sections, preparation, fermentation, and finishing. The preparation section includes microbial culture and mixing. A bed-type SSF bioreactor was suitable for the two-stage solid-state fermentation. Substrate drying, pulverizing, and packaging are included in the finishing section for industrial production.

10.1128/mSystems.00501-19.6 FIG S6 Industrial process flowsheet for the production of FMS based on a bed-type fermentation process. Download FIG S6, TIF file, 1.6 MB. Copyright © 2020 Wang et al. 2020 Wang et al. This content is distributed under the terms of the Creative Commons Attribution 4.0 International license.

## DISCUSSION

In the present study, a novel two-stage SSF method was used to effectively and efficiently alter the physicochemical features, microbiota, and metabolic features of MS. The ANF concentration and pH in the FMS decreased over time, whereas the levels of SP and AP increased. *Bacillus* spp., *Enterococcus* spp., and *Pseudomonas* spp. became the dominant genera and dynamically affected various metabolic pathways during two-stage SSF. Network analysis revealed the relationships of the microbial community with physicochemical features and metabolic functions. Weaned piglets were used as an *in vivo* model to further test the FMS in terms of growth performance, nutrient digestibility, and anti-inflammatory properties.

The glycinin, beta-conglycinin, and TI in MS were dramatically degraded during first-stage fermentation. Many proteases were secreted by B. subtilis during aerobic fermentation, such as aminopeptidases, serine endopeptidases, metalloproteinases, and neutral proteases (Fig. S1E), which are able to decompose proteins, including TI (7). Thus, the hydrolytic effects of the proteases secreted by B. subtilis resulted in a decrease in antigenic protein and TI levels in FMS. Interestingly, further decomposition of antigenic proteins and TI occurred in second-stage fermentation, perhaps due to the low pH and structural changes caused by *Lactobacillus* spp. (8). The increase in xylanase and cellulase activities might be attributed to the decrease in NDF content (Fig. S1F and G). Other enzymes from B. subtilis, such as amylase and phytase, can cause degradation of ANFs (13). Glycinin and beta-conglycinin are two major antigenic proteins that account for approximately 75% of the total soybean proteins and are responsible for allergic reactions (14, 15). TI may hinder digestive functions (2). Additionally, high levels of NDF, phytase, and amylose may interfere with nutrition utilization (16). Therefore, the lower antigenic protein, TI, NDF, phytase, and amylose contents indicate that FMS may have higher nutrient digestibility and lower nutrient emissions than does nonfermented MS.

The CP and SP contents of FMS tended to be higher than those of unfermented MS (*P < *0.05), which is consistent with previous experimental research (17–19). The increase in CP content may be attributed to the loss of dry matter during fermentation (20). A rapid increase in SP content was observed in the study (*P < *0.05). Shi et al. reported that the increase in SP content may be attributed to the digestion of large proteins during fermentation (21). An increased amount of SPs in FMS in the present study might also be due to the digestion of large peptides, especially antigenic proteins in FMS, by proteases from the probiotics. SPs are considered to have antioxidant properties (22), immunoregulatory functions (23), and desirable digestibility (11). Thus, the increased SP content indicated the functional properties of FMS. Additionally, the high LA content and low pH of FMS are essential for pathogen inhibition, feed preservation, and feed intake enhancement (10).

The protein profile obtained by sodium dodecyl sulfate-polyacrylamide gel electrophoresis (SDS-PAGE) revealed that the proteins were degraded in a time-dependent manner, which was consistent with results from previous studies on SBM fermentation by fungi or bacteria (24, 25). Interestingly, the protein profile at 12 h was markedly different from that at 24 h, which suggested that the degradation of proteins mostly occurred during the 12- to 24-h period. To further identify the proteins in FMS, 2DE was applied. The results demonstrated that a considerable number of proteins, especially glycinin, beta-conglycinin, and TI, were greatly degraded to SPs during SSF using B. subtilis and E. faecium. Overall, these SDS-PAGE and 2DE results further confirmed the changes in protein profiles and were generally consistent with the chemical analysis of MS during the fermentation process.

In contrast to MS, FMS exhibited small, cracked structures and large holes. The change in the surface structure of MS after fermentation may be associated with extracellular enzymes (especially protease, amylase, and carbohydrase) secreted during the process. The cracked and porous structure may provide increased access to enzymes for nutrient hydrolysis and may make the substrates considerably easier to utilize (26), suggesting that FMS had higher digestibility than MS. Additionally, Tang et al. reported that smaller protein aggregates may result in a higher solubility (27). Zhao et al. found that soybean proteins with loose networks and diffuse structures have higher emulsification activity and solubility (28). Thus, the physicochemical properties of MS may also have been affected by the changed microstructure in this study.

High-throughput sequencing was first applied to analyze the changes in microbial community structure and metabolic functions during the fermentation process. The decrease in OTU number during first-stage fermentation suggested that B. subtilis proliferated, inhibited other bacteria, and became the dominant bacterium. In contrast, the OTU number increased during anaerobic fermentation, which may be associated with inoculation with E. faecium and the growth of some other LA bacteria. The main phyla (*Cyanobacteria*, *Proteobacteria,* and *Firmicutes*) found in the present study were also obtained in some other studies related to SSF (29, 30).

*Bacillus* spp., *Enterococcus* spp., and *Pseudomonas* spp. were identified as the core genera during two-stage SSF. Members of the heat-tolerant genus *Bacillus* are effective at degrading proteins and cellulose during SSF due to their strong hydrolytic abilities (29). *Enterococcus* is a mesophilic genus whose members generate LA (30). *Pseudomonas* spp. have been investigated as biocontrol bacteria to promote fermentation quality in the maturation period (31). Thus, these dominant genera indicated a selected community categorized by typical large-molecule catabolism characteristics and the capacity to produce LA, achieved by the addition of the two inocula. The evolution of bacterial structure during the process demonstrated that the artificially added inocula not only increased the number of added bacteria but also boosted some other functional bacteria that could develop a form of symbiosis with the inocula.

The results of KEGG level 1 to 3 gene function analysis were generally consistent. As fermentation progressed, the abundances of both carbohydrate and amino acid metabolism genes gradually increased. Metabolism of cellulose and hemicellulose can produce many compounds that support bacterial growth (32). Amino acids are also an energy and carbon source for bacteria (33). These results indicated that the degradation of large carbohydrate and proteins resulted in increased levels of saccharides, SPs, and amino acids, which could be utilized by the microbiota in FMS. The gene abundances of transporters, ABC transporters, and transcription factors increased during fermentation. These metabolic functions were associated with compound production and membrane transport, suggesting the mechanism of enzyme synthesis by and activity of the core bacteria in FMS. In contrast, cellular processes and signaling and energy metabolism were inhibited by the core genera, indicating that these genes may be involved in native bacterial gene functions of MS. Although the addition of B. subtilis inhibited the growth of other MS-native microbes, it did not decrease the abundances of enzyme families, which were maintained at approximately 10%. Enzyme families are important for complex biopolymer catabolism (34). This evidence revealed the strong enzyme secretion ability of B. subtilis. Additionally, the differences in the abundances of genes involved in translation and nucleotide metabolism between the aerobic and anaerobic stages suggested the different metabolic roles of *Bacillus* spp. and *Enterococcus* spp.

AP is critical for cell growth and development (35). During the first 24 h of fermentation, most native genera in MF had a negative relationship with AP, suggesting that they may utilize AP for growth. *Bacillus* spp. were positively related to AP, which may be due to the higher rate of phytate phosphorus degradation than utilization by B. subtilis. In addition, *Bacillus* spp. were negatively related to most of the native microbes, revealing that AP strongly affected the growth of these bacteria and that *Bacillus* competed with other genera. *Bacillus* spp. participated in the transformation of large proteins and glycinin to SPs and were thus positively related to SP and negatively related to glycinin. Meanwhile, *Bacillus* spp. increased the pH of the fermentation system; thus, they were positively related to pH. In the second anaerobic fermentation stage, *Enterococcus* spp. were positively related to LA, indicating that the major function of *Enterococcus* spp. was LA generation. Intriguingly, *Bacillus* spp. were negatively related to SP, while the amount of SP continued to increase. This phenomenon suggested that although the abundance of *Bacillus* spp. decreased during the second stage, their metabolic activities, such as those performed via enzymes and bacteriocin, might continue to increase the amount of SPs and inhibit the growth of Facklamia spp. and *Enterococcaceae*.

Various bacteria were correlated with different metabolic pathways, revealing that multiple metabolic pathways were active during the two stages of the SSF. Two inoculated bacteria were enriched in environmental information processing and cellular processes throughout fermentation. These metabolic functions allowed the bacteria to grow, proliferate, and respond to the environment (36). The results demonstrated the superior adaptation of the inocula in response to fermentation. Additionally, under aerobic conditions, some feed-native genera were positively related to genetic information processing and organic systems, whereas members of *Bacillus* spp. were negatively involved with these metabolic pathways. This evidence suggests that *Bacillus* spp. might inhibit feed-native bacteria by suppressing genetic information processing and organic systems.

Both 16S rRNA analysis and PICRUSt predictions are dependent on the database, which may cause deviation; thus, real-time quantitative PCR was applied to further assess the accuracy of these results. The results of the bacterial assessment were highly consistent with the 16S rRNA analysis. Although the functional gene expression results at some time points were discrepant, the general metabolic assessments were similar to the PICRUSt prediction. The results of quantitative PCR (qPCR) further verified the bacterial communities and functional genes present during fermentation.

Weaned piglets were used as an *in vivo* model to evaluate the nutrient utilization and anti-inflammatory effects of FMS. FMS was beneficial for piglet growth performance, which may be due to the high digestibility of FMS and probiotic roles of the inocula. The increased CP and TP digestibility of FMS suggested the ease of utilization of the structures of FMS and the low N and P emissions. The improved villus height-to-crypt depth ratio indicated that FMS improved piglet gut morphology. Intestinal mucosal immune responses to glycinin were enhanced by high levels of IL-4 and IL-6 (37). The MAPK/NF-κB pathway is critical for regulating inflammatory genes (38). The reduced levels of IL-4 and IL-6 and phosphorylation of p38, IκB-α, and p65 in the jejunum further confirmed that FMS reduced the immune disorder that might be caused by antigenic proteins.

In summary, this study provides a novel method for improving the nutritional quality of MS and provides a basis for demonstrating that the inoculated microbes dynamically change the physicochemical features, microbiota, and metabolic functions during two-stage SSF, which could serve as a valuable resource for industrial feed-based practices and metabolomic research on SSF systems. Further studies should focus on the use of additional enzymes during first-stage fermentation and inoculation with other LA bacteria during second-stage fermentation to further reduce the ANF content of FMS and produce various types of organic acids.

## MATERIALS AND METHODS

### Microorganisms and enzymes.

B. subtilis CW4 (NCBI accession no. MH885533) was obtained from a traditional fermented food (pickled vegetables) and was selected by using a soybean antigenic protein screening plate. E. faecium CWEF (NCBI accession no. MN038173) was isolated from the gut of a healthy pig. Both B. subtilis and E. faecium are government-authorized probiotics in China.

S. cerevisiae (CGMCC 2.3973), *L. plantarum* (CGMCC 1.16089), Lactobacillus casei (CGMCC 1.8727), and neutral protease from *Bacillus* spp. (P3111; Sigma-Aldrich Corp., St. Louis, MO, USA) were purchased to compare their effects on protein degradation and LA production during different fermentation processes.

### Preparation of fermented mixed substrates.

For inoculation, B. subtilis and E. faecium were cultured in Luria broth and de Man, Rogosa, and Sharpe liquid medium at 37°C for 10 h and 18 h, respectively. The fermented substrates were finely mixed in a corn/SBM/rDDGS ratio of 2:2:1 (total, 200 g). Then, the MS was placed in a 500-ml Erlenmeyer flask and supplied with water to achieve a 35% moisture concentration. Next, a sterile membrane was added to the Erlenmeyer flask.

The moist MS was inoculated with B. subtilis (10^7^ CFU/g) and fermented at 37°C for 24 h (first-stage aerobic fermentation). Then, the sterile membrane was removed, and each Erlenmeyer flask was supplied with E. faecium (10^7^ CFU/g). The mouth of the Erlenmeyer flask was sealed with a rubber plug for the second-stage anaerobic fermentation at 37°C. All samples were set up in quadruplicate. Moist samples (approximately 100 g) at 0 h, 12 h, 24 h, and 48 h were collected to determine the numbers of microorganisms and microbial metabolites and for 16S rRNA gene sequencing, and the remaining samples were dried at 60°C for 24 h, cooled, ground, and subjected to physicochemical analysis, SDS-PAGE, and 2DE.

### Microorganisms and metabolites.

The pH and microbiological counts at different fermentation times were analyzed as described by Wang et al. (39). Xylanase and cellulase activities were analyzed by the dinitrosalicylic acid method reported by Wongputtisin et al. (40). The activity of neutral protease was detected as mentioned by Ueda et al. (41).

### Chemical analysis.

Samples at 0 h, 12 h, 24 h, and 48 h were obtained for analysis of the dry matter, CP, NDF, acid detergent fiber (ADF), total starch, amylose, ash, calcium (Ca), TP, and AP contents using AOAC International guidelines (42). Total organic carbon (TOC) and nitrogen were measured on a Vario EL cube (Elementar Americas, Inc., Hanau, Germany). The TCA-SP, free amino acid, and SP contents of the sample were analyzed as reported by Ovissipour et al. (43). The phytate phosphorus content was measured according to the method described by Thompson and Erdman (44). The concentrations of antigenic proteins in MS and FMS were analyzed using an enzyme-linked immunosorbent assay (ELISA) kit (Jiangsu Meibiao Biological Technology Co., Ltd., Jiangsu, People’s Republic of China), according to the manufacturer’s protocol.

### Microscopic inspection.

Changes in the physical properties of the substrates before and after fermentation were examined by SEM according to the protocol of the Electronic Microscopy Center of Zhejiang University. The microstructures of MS and FMS were observed using a field-emission scanning electron microscope (KYKY-EM3200, China) at ×100, ×1,500, and ×5,000 magnifications.

### Electrophoresis.

The protein profile of MS was obtained as described by Zhang et al. (45). Samples (25 g) at 0 h, 12 h, 24 h, and 48 h were soaked overnight in 50 ml of 50 mM acetate buffer (pH 5.0) containing 5 μg/ml protease inhibitor (Roche, Switzerland) at 4°C. The suspension was centrifuged and filtered (Millipore, USA). The soluble protein samples were concentrated by ultrafiltration (cutoff, 10 kDa; Millipore). Then, 10 μl of 20 mg/ml sodium deoxycholate was added to 1 ml of the prepared protein solution, vortexed, and kept for 30 min on ice. TCA was added to achieve a concentration of 12% (wt/vol), and the mixture was incubated for 1 h at 4°C. The precipitate was washed, centrifuged, and solubilized in lysis buffer (GE Healthcare, USA). The protein concentration was determined using the Bio-Rad protein assay (USA), according to the manufacturer’s protocols.

For SDS-PAGE, 12% polyacrylamide separating gels were used for electrophoresis. Approximately 5 μg of protein sample was placed in each well, and the sample was separated at 55 mV for 200 min. The gel was stained with Coomassie brilliant blue (CBB) R-250 (Bio-Rad, USA) for 60 min and destained with eluent.

For 2DE, 400 μg of protein was placed onto analytical and preparative gels. Isoelectric focusing (IEF) was conducted using the Ettan IPGphor IEF system (GE Amersham) and pH 3- to 10-immobilized pH gradient (IPG) strips (13 cm, nonlinear; GE Healthcare). The IPG strips were rehydrated in rehydration buffer containing the protein samples. Equilibration buffer was used to equilibrate the gel strips. Then, the strips were subjected to 2DE after transfer onto 12.5% SDS-polyacrylamide gels. Protein spot identification was conducted as reported in reference 9.

### DNA extraction, Illumina MiSeq sequencing, and metabolic function prediction.

Total DNA was extracted from 16 samples using the E.Z.N.A. soil DNA kit (Omega Bio-Tek, Norcross, GA, USA). A NanoDrop 2000 UV-vis spectrophotometer (Thermo Scientific, Wilmington, DE, USA) and 1% agarose gel electrophoresis were used to analyze DNA content and quality.

The V3-V4 gene regions of the bacterial 16S rRNA gene were amplified with primers 338F (5′-ACTCCTACGGGAGGCAGCAG-3′) and 806R (5′-GGACTACHVGGGTWTCTAAT-3′). PCR was conducted as follows: 3 min of denaturation at 95°C; 27 cycles of 30 s at 95°C, 30 s for annealing at 55°C, and 45 s for elongation at 72°C; and a final extension at 72°C for 10 min. The AxyPrep DNA gel extraction kit (Axygen Biosciences, Union City, CA, USA) and QuantiFluor-ST instrument (Promega, USA) were used to further extract, purify, and quantify the PCR products. The MiSeq platform (Shanghai Majorbio Biopharm Technology Co., Ltd.) was used to describe the bacterial community based on the gene segment from the V3-V4 portion of the 16S rRNA gene. Subsequently, raw Illumina FASTQ files were demultiplexed, quality filtered, and analyzed using Quantitative Insights into Microbial Ecology (QIIME v1.9.1). Raw fastq files were quality filtered by Trichromatic and merged by FLASH. OTUs were clustered with 97% similarity cutoff using UPARSE (version 7.1). The taxonomy of each 16S rRNA gene sequence was analyzed using the RDP Classifier algorithm (http://rdp.cme.msu.edu/) against the Greengenes 16S rRNA database using a confidence threshold of 70%. The assembled MiSeq sequences were submitted to the NCBI’s Sequence Read Archive (SRA BioProject no. PRJNA551719) for open access. Estimates of diversity values for these samples using the Chao1, Shannon, and Simpson indexes for diversity estimation were calculated by rarefaction analysis. Good’s coverage analysis was also performed. PCA and cluster analysis with the Ward method were conducted using the Web server tool METAGENassist based on unweighted UniFrac distances.

The main differentially abundant genera were selected by the LEfSe method (https://huttenhower.sph.harvard.edu/galaxy/). To predict metabolic genes during the process, PICRUSt (https://huttenhower.sph.harvard.edu/galaxy/) was applied to obtain a functional profile from the 16S rRNA data. Prior to metagenome prediction, the OTUs of 16S rRNA sequences were analyzed using PICRUSt. PICRUSt and KEGG were used to obtain functions for the genes that were predicted to be present in the samples and to assign the genes into metabolic pathways.

Network analysis was conducted using Cytoscape, and the nonparametric Spearman correlation coefficient was greater than 0.8.

### Quantitative analysis of commensal bacterial and functional genes.

The extracted DNA was used to quantify the bacterial content and metabolic functions of the FMS system. DNA was extracted, and quantitative PCR for 16S rRNA genes and functional genes was performed with SYBR green master mix (Roche, Mannheim, Germany) using a StepOnePlus real-time PCR system (Applied Biosystems, Foster City, CA, USA); the results were normalized to the total bacterial DNA content. The gene-specific primers for the qPCR are listed in Table S2.

10.1128/mSystems.00501-19.8 TABLE S2 qPCR primers for bacterial and functional gene tests. Download Table S2, DOCX file, 0.1 MB. Copyright © 2020 Wang et al. 2020 Wang et al. This content is distributed under the terms of the Creative Commons Attribution 4.0 International license.

### Animal management, nutrient digestibility, and intestinal morphology assessment.

The *in vivo* experimental design is presented in Fig. 1Ab. The experimental protocols were approved by the Institutional Animal Care and Use Committee of Zhejiang University. Briefly, a 21-day experiment was conducted with 144 weaned pigs (Duroc × Large white × Yorkshire) with body weights of 9.00 ± 0.65 kg, which were randomly allocated to the control group or the 10% FMS supplementation group. Each group contained 6 pens, and each pen had 12 piglets. Piglets were fed four times a day, and they had free access to water and feed. The ingredients and nutrient content of the diets are presented in Table S3. Nutrient digestibility was calculated as described by Wang et al. (46). The middle jejunum of the piglets was harvested, fixed in 4% paraformaldehyde, and embedded in paraffin. Sections of 5-μm thickness were deparaffinized in xylene and stained with hematoxylin and eosin (H&E). Images were obtained using a DM3000 microscope (Leica Microsystems, Wetzlar, Germany). The villous height and crypt depth of the jejunum were measured with Image-Pro software (Media Cybernetics, Rockville, MD).

10.1128/mSystems.00501-19.9 TABLE S3 Ingredient compositions and nutrient concentrations in the experimental diets (as-fed basis). Download Table S3, DOCX file, 0.1 MB. Copyright © 2020 Wang et al. 2020 Wang et al. This content is distributed under the terms of the Creative Commons Attribution 4.0 International license.

### Real-time quantitative PCR and Western blot analysis.

Quantitative PCR of cytokines was conducted as described above. For Western blotting, total protein extracts of scraped jejunal mucosa or cells were harvested using the Total protein extraction kit (KeyGen BioTECH, Nanjing, China). Equivalent amounts of protein were separated by SDS-PAGE and electroblotted onto polyvinylidene difluoride (PVDF) membranes (Millipore, Bedford, MA, USA), followed by blocking with 5% fat-free milk. Then, membranes were incubated overnight at 4°C with primary antibodies, including p-p38, p-IκB, p-p65, and β-actin antibodies (ABcom, China). After washing with Tris-buffered saline with Tween 20 (TBST), membranes were incubated with secondary antibodies for 1 h at room temperature. The protein bands were visualized with an electrochemiluminescence (ECL) assay kit (Servicebio, Wuhan, China), and the band intensity was quantified using the ImageJ software.

### Statistical and bioinformatics analyses.

The data were analyzed using the SAS software (version 9.2; SAS, Inc., Chicago, IL, USA). Statistical differences between experimental groups were evaluated by Student’s *t* tests and one-way analysis of variance (ANOVA) with Duncan’s multiple-range test or at least a significant difference test. All data are expressed as the mean and standard deviation (SD). The interquartile range method, followed by quantile normalization within replicates, was performed for data filtering and normalization during the assay. *P* values of ≤0.05 represent a significant difference. For network analyses, the nonparametric Spearman correlation coefficient and significance between bacteria and physicochemical characteristics or bacteria and metabolic functions were calculated using the corplot package of R (R Core Team, 2014). The nonparametric Spearman correlation coefficient, which was greater than 0.8, and significance, which was smaller than 0.05, were selected for further network analysis. The heatmap package of R (R Core Team, 2014) was applied to generate heat maps of genera and L3 predicted microbial gene functions. Bar plots were generated in GraphPad Prism 7 (San Diego, CA, USA). Multiple-testing corrections of distinguished species and predicted metabolic functions during fermentation were employed using Welch’s test and the Benjamini-Hochberg false-discovery rate (FDR) method for statistical analysis of metagenomic profiles (STAMP version 2.1.3).

## References

[1] Feng J, Liu X, Xu ZR, Lu YP, Liu YY 2007 Effect of fermented soybean meal on intestinal morphology and digestive enzyme activities in weaned piglets. Dig Dis Sci 52:1845–1850. doi:10.1007/s10620-006-9705-0. 17410452

[2] Li DF, Nelssen JL, Reddy PG, Blecha F, Hancock JD, Allee GL, Goodband RD, Klemm RD 1990 Transient hypersensitivity to soybean-meal in the early-weaned pig. J Anim Sci 68:1790–1799. doi:10.2527/1990.6861790x. 2384373

[3] Flavin DF 1982 The effects of soybean trypsin-inhibitors on the pancreas of animals and man–a review. Vet Hum Toxicol 24:25–28. 7036513

[4] Woyengo TA, Nyachoti CM 2013 Review: anti-nutritional effects of phytic acid in diets for pigs and poultry–current knowledge and directions for future research. Can J Anim Sci 93:9–21. doi:10.4141/cjas2012-017.

[5] Jha R, Woyengo TA, Li J, Bedford MR, Vasanthan T, Zijlstra RT 2015 Enzymes enhance degradation of the fiber-starch-protein matrix of distillers dried grains with solubles as revealed by a porcine in vitro fermentation model and microscopy. J Anim Sci 93:1039–1051. doi:10.2527/jas.2014-7910. 26020881

[6] Song YS, Perez VG, Pettigrew JE, Martinez-Villaluenga C, de Mejia EG 2010 Fermentation of soybean meal and its inclusion in diets for newly weaned pigs reduced diarrhea and measures of immunoreactivity in the plasma. Anim Feed Sci Tech 159:41–49. doi:10.1016/j.anifeedsci.2010.04.011.

[7] Hu JK, Lu WQ, Wang CL, Zhu RH, Qiao JY 2008 Characteristics of solid-state fermented feed and its effects on performance and nutrient digestibility in growing-finishing pigs. Asian Australas J Anim Sci 21:1635–1641. doi:10.5713/ajas.2008.80032.

[8] Chi CH, Cho SJ 2016 Improvement of bioactivity of soybean meal by solid-state fermentation with *Bacillus amyloliquefaciens* versus *Lactobacillus* spp. and *Saccharomyces cerevisiae*. LWT Food Sci Technol 68:619–625. doi:10.1016/j.lwt.2015.12.002.

[9] Seo SH, Cho SJ 2016 Changes in allergenic and antinutritional protein profiles of soybean meal during solid-state fermentation with *Bacillus subtilis*. LWT Food Sci Technol 70:208–212. doi:10.1016/j.lwt.2016.02.035.

[10] Missotten JAM, Michiels J, Degroote J, De Smet S 2015 Fermented liquid feed for pigs: an ancient technique for the future. J Anim Sci Biotechnol 6:4. doi:10.1186/2049-1891-6-4. 25838899 PMC4383217

[11] Wang C, Su WF, Zhang Y, Hao LH, Wang FQ, Lu ZQ, Zhao J, Liu XL, Wang YZ 2018 Solid-state fermentation of distilled dried grain with solubles with probiotics for degrading lignocellulose and upgrading nutrient utilization. AMB Express 8:188. doi:10.1186/s13568-018-0715-z. 30478751 PMC6261088

[12] Zhao C, Dong H, Zhang Y, Li Y 2019 Discovery of potential genes contributing to the biosynthesis of short-chain fatty acids and lactate in gut microbiota from systematic investigation in E. coli. NPJ Biofilms Microbiomes 5:19. doi:10.1038/s41522-019-0092-7. 31312512 PMC6626047

[13] Simonen M, Palva I 1993 Protein secretion in Bacillus species. Microbiol Rev 57:109–137. 8464403 10.1128/mr.57.1.109-137.1993 PMC372902

[14] Natarajan S, Xu CP, Caperna TJ, Garrett WA 2005 Comparison of protein solubilization methods suitable for proteomic analysis of soybean seed proteins. Anal Biochem 342:214–220. doi:10.1016/j.ab.2005.04.046. 15953580

[15] Maruyama N, Adachi M, Takahashi K, Yagasaki K, Kohno M, Takenaka Y, Okuda E, Nakagawa S, Mikami B, Utsumi S 2001 Crystal structures of recombinant and native soybean beta-conglycinin beta homotrimers. Eur J Biochem 268:3595–3604. doi:10.1046/j.1432-1327.2001.02268.x. 11422391

[16] Zhang L, Ouyang Y, Li HT, Shen L, Ni YQ, Fang QC, Wu GY, Qian LL, Xiao YF, Zhang J, Yin PY, Panagiotou G, Xu GW, Ye JP, Jia WP 2019 Metabolic phenotypes and the gut microbiota in response to dietary resistant starch type 2 in normal-weight subjects: a randomized crossover trial. Sci Rep 9:4736. doi:10.1038/s41598-018-38216-9. 30894560 PMC6426958

[17] Teng D, Gao M, Yang Y, Liu B, Tian Z, Wang J 2012 Bio-modification of soybean meal with Bacillus subtilis or Aspergillus oryzae. Biocatal Agric Biotechnol 1:32–38. doi:10.1016/j.bcab.2011.08.005.

[18] Cervantes-Pahm SK, Stein HH 2010 Ileal digestibility of amino acids in conventional, fermented, and enzyme-treated soybean meal and in soy protein isolate, fish meal, and casein fed to weanling pigs. J Anim Sci 88:2674–2683. doi:10.2527/jas.2009-2677. 20407072

[19] Frias J, Song YS, Martinez-Villaluenga C, De Mejia EG, Vidal-Valverde C 2008 Immunoreactivity and amino acid content of fermented soybean products. J Agric Food Chem 56:99–105. doi:10.1021/jf072177j. 18072744

[20] Rozan P, Villaum C, Bau HM, Schwertz A, Nicolas JP, Mejean L 1996 Detoxication of rapeseed meal by *Rhizopus oligosporus* sp.-T3: a first step towards rapeseed protein concentrate. Int J Food Sci Tech 31:85–90. doi:10.1111/j.1365-2621.1996.17-315.x.

[21] Shi CY, Zhang Y, Lu ZQ, Wang YZ 2017 Solid-state fermentation of corn-soybean meal mixed feed with *Bacillus subtilis* and *Enterococcus faecium* for degrading antinutritional factors and enhancing nutritional value. J Anim Sci Biotechnol 8:50. doi:10.1186/s40104-017-0184-2. 28603613 PMC5465572

[22] Sarmadi BH, Ismail A 2010 Antioxidative peptides from food proteins: a review. Peptides 31:1949–1956. doi:10.1016/j.peptides.2010.06.020. 20600423

[23] Yi HB, Hu WY, Chen S, Lu ZQ, Wang YZ 2017 Cathelicidin-WA improves intestinal epithelial barrier function and enhances host defense against enterohemorrhagic *Escherichia coli* O157:H7 Infection. J Immunol 198:1696–1705. doi:10.4049/jimmunol.1601221. 28062699

[24] Kook M-C, Cho S-C, Hong Y-H, Park H 2014 *Bacillus subtilis* Fermentation for enhancement of feed nutritive value of soybean meal. J Appl Biol Chem 57:183–188. doi:10.3839/jabc.2014.030.

[25] Hong KJ, Lee CH, Kim SW 2004 *Aspergillus oryzae* GB-107 fermentation improves nutritional quality of food soybeans and feed soybean meals. J Med Food 7:430–435. doi:10.1089/jmf.2004.7.430. 15671685

[26] Zheng L, Li D, Li ZL, Kang LN, Jiang YY, Liu XY, Chi YP, Li YQ, Wang JH 2017 Effects of bacillus fermentation on the protein microstructure and anti-nutritional factors of soybean meal. Lett Appl Microbiol 65:520–526. doi:10.1111/lam.12806. 28975646

[27] Tang CH, Wang XY, Yang XQ, Li L 2009 Formation of soluble aggregates from insoluble commercial soy protein isolate by means of ultrasonic treatment and their gelling properties. J Food Eng 92:432–437. doi:10.1016/j.jfoodeng.2008.12.017.

[28] Zhao XY, Zhu HT, Zhang BW, Chen J, Ao Q, Wang XY 2015 XRD, SEM, and XPS analysis of soybean protein powders obtained through extraction involving reverse micelles. J Am Oil Chem Soc 92:975–983. doi:10.1007/s11746-015-2657-9.

[29] de Gannes V, Eudoxie G, Hickey WJ 2013 Prokaryotic successions and diversity in composts as revealed by 454-pyrosequencing. Bioresour Technol 133:573–580. doi:10.1016/j.biortech.2013.01.138. 23475177

[30] Yuan SF, Hsu TC, Wang CA, Jang MF, Kuo YC, Alper HS, Guo GL, Hwang WS 2018 Production of optically pure l(+)-lactic acid from waste plywood chips using an isolated thermotolerant *Enterococcus faecalis* SI at a pilot scale. J Ind Microbiol Biotechnol 45:961–970. doi:10.1007/s10295-018-2078-5. 30182264

[31] Ventorino V, Parillo R, Testa A, Viscardi S, Espresso F, Pepe O 2016 Chestnut green waste composting for sustainable forest management: microbiota dynamics and impact on plant disease control. J Environ Manage 166:168–177. doi:10.1016/j.jenvman.2015.10.018. 26496847

[32] Toledo M, Gutiérrez MC, Siles JA, García-Olmo J, Martín MA 2017 Chemometric analysis and NIR spectroscopy to evaluate odorous impact during the composting of different raw materials. J Clean Prod 167:154–162. doi:10.1016/j.jclepro.2017.08.163.

[33] López-González JA, Suárez-Estrella F, Vargas-García MC, López MJ, Jurado MM, Moreno J 2015 Dynamics of bacterial microbiota during lignocellulosic waste composting: studies upon its structure, functionality and biodiversity. Bioresour Technol 175:406–416. doi:10.1016/j.biortech.2014.10.123. 25459849

[34] Kausar H, Sariah M, Saud HM, Alam M, Ismail M 2011 Isolation and screening of potential actinobacteria for rapid composting of rice straw. Biodegradation 22:367–375. doi:10.1007/s10532-010-9407-3. 20803236

[35] Sánchez ÓJ, Ospina DA, Montoya S 2017 Compost supplementation with nutrients and microorganisms in composting processes. Waste Manag 69:136–153. doi:10.1016/j.wasman.2017.08.012. 28823698

[36] Wei HW, Wang LH, Hassan M, Xie B 2018 Succession of the functional microbial communities and the metabolic functions in maize straw composting processes. Bioresour Technol 256:333–341. doi:10.1016/j.biortech.2018.02.050. 29459320

[37] Sun P, Li D, Li Z, Dong B, Wang F 2008 Effects of glycinin on IgE-mediated increase of mast cell numbers and histamine release in the small intestine. J Nutr Biochem 19:627–633. doi:10.1016/j.jnutbio.2007.08.007. 18280135

[38] Fan Y, Wang J, Wei L, He B, Wang C, Wang B 2011 Iron deficiency activates pro-inflammatory signaling in macrophages and foam cells via the p38 MAPK-NF-κB pathway. Int J Cardiol 152:49–55. doi:10.1016/j.ijcard.2010.07.005. 20674992

[39] Wang J, Han Y, Zhao JZ, Zhou ZJ, Fan H 2017 Consuming fermented distillers’ dried grains with solubles (DDGS) feed reveals a shift in the faecal microbiota of growing and fattening pigs using 454 pyrosequencing. J Integr Agr 16:900–910. doi:10.1016/S2095-3119(16)61523-X.

[40] Wongputtisin P, Khanongnuch C, Khongbantad W, Niamsup P, Lumyong S 2012 Screening and selection of *Bacillus* spp. for fermented corticate soybean meal production. J Appl Microbiol 113:798–806. doi:10.1111/j.1365-2672.2012.05395.x. 22788990

[41] Ueda M, Kubo T, Miyatake K, Nakamura T 2007 Purification and characterization of fibrinolytic alkaline protease from *Fusarium* spp. BLB. Appl Microbiol Biotechnol 74:331–338. doi:10.1007/s00253-006-0621-1. 17221202

[42] AOAC International. 2010 Official methods of analysis of AOAC International, current through revision 4, 18th ed. AOAC International, Gaithersburg, MD.

[43] Ovissipour M, Abedian A, Motamedzadegan A, Rasco B, Safari R, Shahiri H 2009 The effect of enzymatic hydrolysis time and temperature on the properties of protein hydrolysates from Persian sturgeon (Acipenser persicus) viscera. Food Chem 115:238–242. doi:10.1016/j.foodchem.2008.12.013.

[44] Thompson DB, Erdman JW 1982 Phytic acid determination in soybeans. J Food Sci 47:513–517. doi:10.1111/j.1365-2621.1982.tb10114.x.

[45] Zhang B, Guan ZB, Cao Y, Xie GF, Lu J 2012 Secretome of *Aspergillus oryzae* in Shaoxing rice wine koji. Int J Food Microbiol 155:113–119. doi:10.1016/j.ijfoodmicro.2012.01.014. 22341915

[46] Wang C, Shi C, Lu Z, Zhang Y, Wang Y 2018 Effects of supplementing sow diets with fermented corn and soybean meal mixed feed during lactation on the performance of sows and progeny. J Anim Sci 96:306–307. doi:10.1093/jas/sky404.674. 29378011 PMC6140954

