# Supplementary figures and images for: Dynamics of the Physicochemical Characteristics, Microbiota, and Metabolic Functions of Soybean Meal and Corn Mixed Substrates during Two-Stage Solid-State Fermentation
Source: mSystems. 2020 Feb 11;5(1):e00501-19. doi: 10.1128/mSystems.00501-19 (PMC7018524; doi:10.1128/mSystems.00501-19)

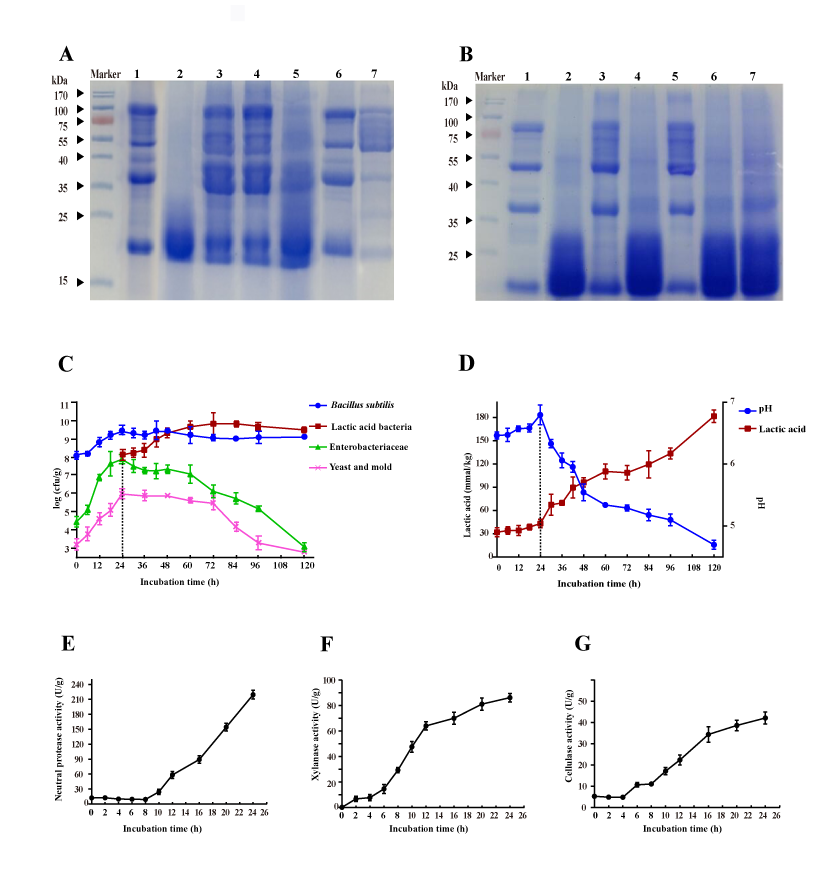

Supplement: FIG S1 [file mSystems.00501-19-sf001.tif]

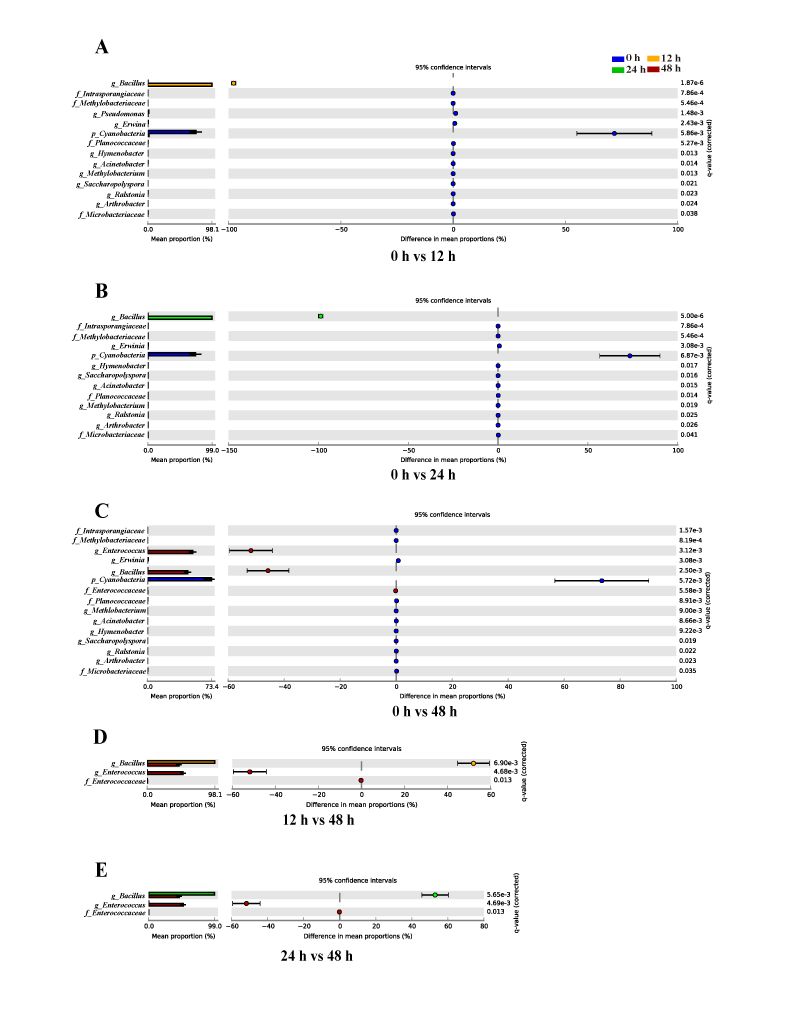

Supplement: FIG S2 [file mSystems.00501-19-sf002.tif]

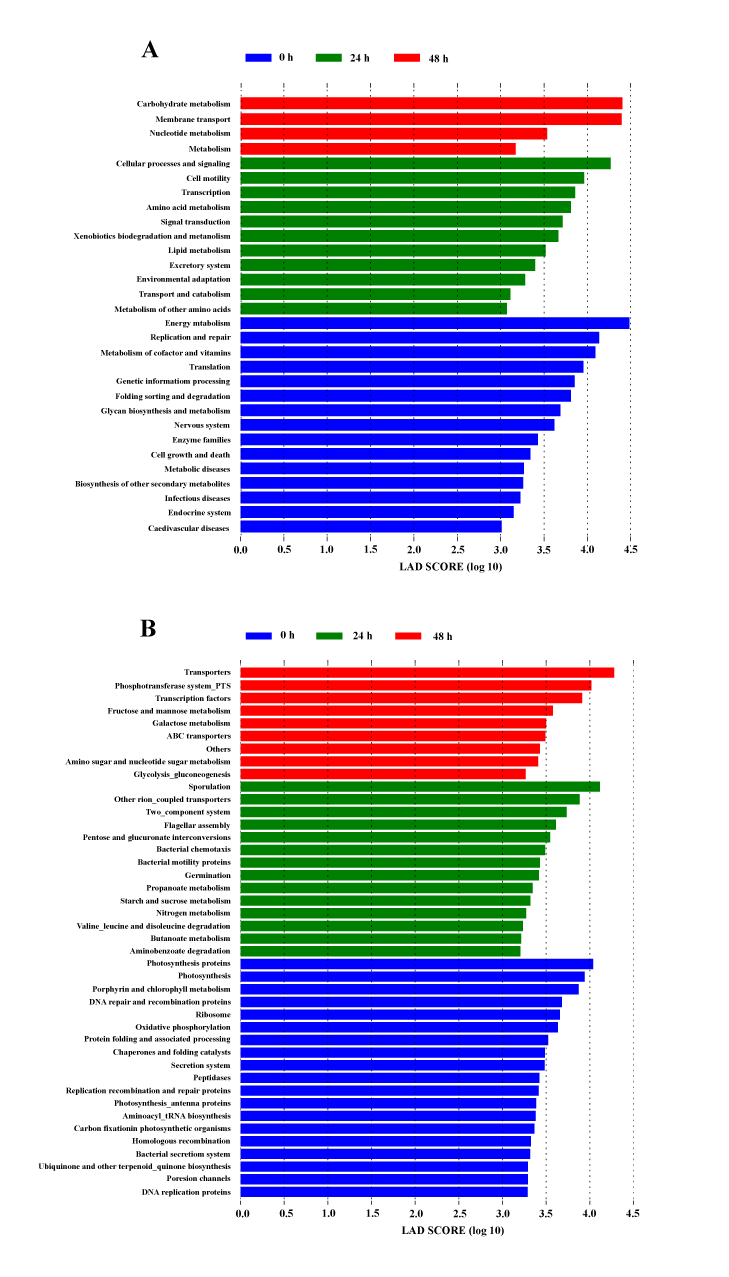

Supplement: FIG S3 [file mSystems.00501-19-sf003.tif]

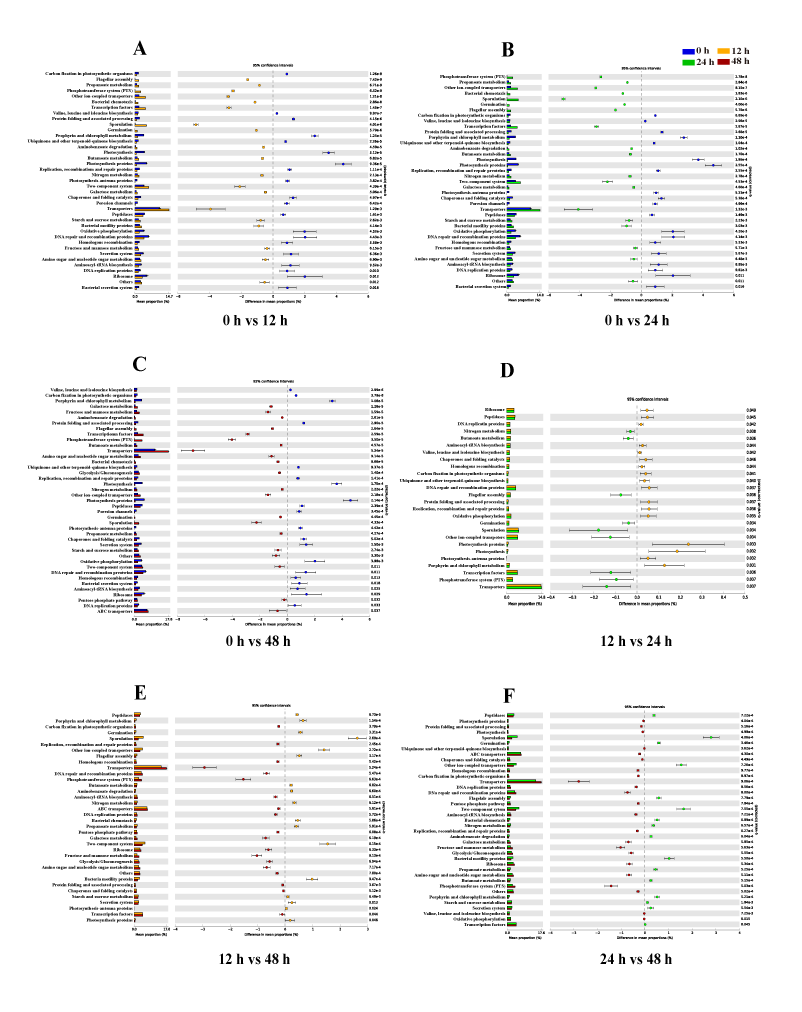

Supplement: FIG S4 [file mSystems.00501-19-sf004.tif]

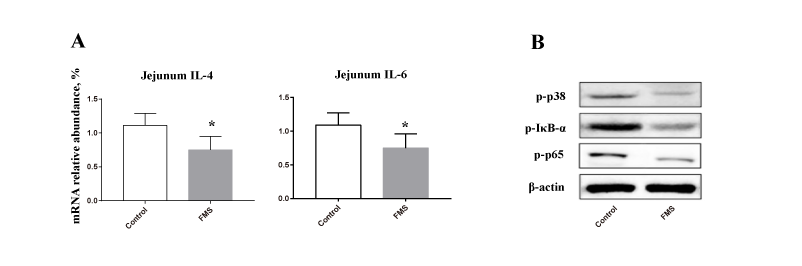

Supplement: FIG S5 [file mSystems.00501-19-sf005.tif]

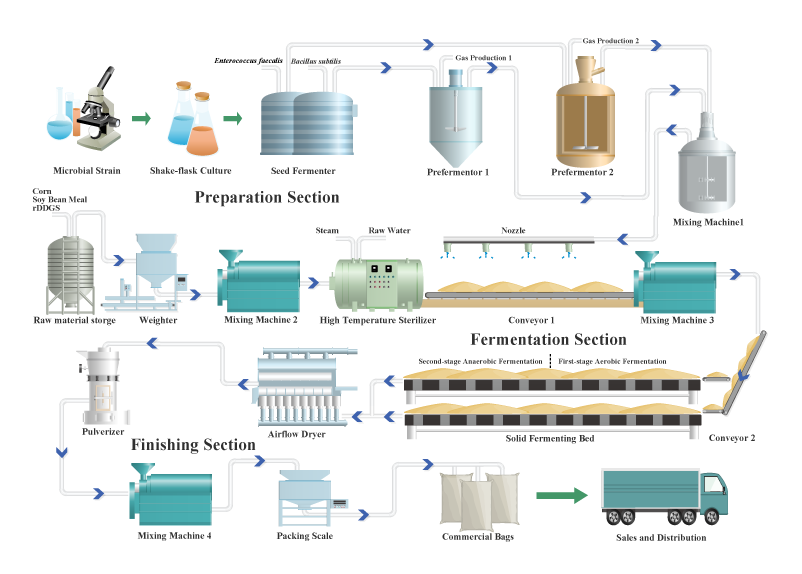

Supplement: FIG S6 [file mSystems.00501-19-sf006.tif]
