# Supplementary material for: Dynamics of the Physicochemical Characteristics, Microbiota, and Metabolic Functions of Soybean Meal and Corn Mixed Substrates during Two-Stage Solid-State Fermentation
Source: mSystems. 2020 Feb 11;5(1):e00501-19. doi: 10.1128/mSystems.00501-19 (PMC7018524; doi:10.1128/mSystems.00501-19)
Supplement: TABLE S1 [file mSystems.00501-19-st001.docx]

**Table S1.**

| Item | N (%) | C (%) | C/N ratio (%) |
| --- | --- | --- | --- |
| Corn | 1.14 | 45.90 | 40.33 |
| SBM | 7.99 | 45.70 | 5.72 |
| Wheat brane | 3.17 | 46.24 | 14.57 |
| Mixed substrate | 4.22 | 45.48 | 10.77 |
