# Supplementary material for: Dynamics of the Physicochemical Characteristics, Microbiota, and Metabolic Functions of Soybean Meal and Corn Mixed Substrates during Two-Stage Solid-State Fermentation
Source: mSystems. 2020 Feb 11;5(1):e00501-19. doi: 10.1128/mSystems.00501-19 (PMC7018524; doi:10.1128/mSystems.00501-19)
Supplement: TABLE S4 [file mSystems.00501-19-st004.docx]

**Table S4.**

| Item | Control | FMS |
| --- | --- | --- |
| Growth performance | | |
| Initial BW, kg | 9.34±0.27 | 9.21±0.21 |
| Final BW, kg | 18.72±0.63 | 19.56±0.95 |
| ADG, g | 378.50±31.39^b^ | 434.33±42.58^a^ |
| ADFI, g | 590.67±26.10 | 625.50±46.08 |
| G: F | 1.57±0.11 | 1.46±0.24 |
| Diarrhea incidence, % | 11.60±2.49^a^ | 7.08±1.87^b^ |
| Nutrient digestibility |  |  |
| Crude protein, % | 79.76±1.68^b^ | 82.63±1.27^a^ |
| Total phosphorus, % | 64.15±0.90^b^ | 74.02±1.31^a^ |
| Jejunal morphology | | |
| Villus height, µm | 360.50±43.15^b^ | 449.17±55.42^a^ |
| Crypt depth, µm | 249.83±30.29 | 235.33±26.44 |
| Villus height: crypt depth | 1.46±0.24^b^ | 1.94±0.39^a^ |
