# Supplementary material for: Dynamics of the Physicochemical Characteristics, Microbiota, and Metabolic Functions of Soybean Meal and Corn Mixed Substrates during Two-Stage Solid-State Fermentation
Source: mSystems. 2020 Feb 11;5(1):e00501-19. doi: 10.1128/mSystems.00501-19 (PMC7018524; doi:10.1128/mSystems.00501-19)
Supplement: TABLE S2 [file mSystems.00501-19-st002.docx]

**Table S2**.

| Item | Primer | Reference |
| --- | --- | --- |
| Bacteria | | |
| Total bacteria-F | ACTCCTACGGGAGGCAGCAG | This study |
| Total bacteria-R | GGACTACHVGGGTWTCTAAT |  |
| Bacillus-F | GCAACGAGCGCAACCCTTGA | This study |
| Bacillus-R | TCATCCCCACCTTCCTCCGGT |  |
| Enterococcus-F | CCCTTATTGTTAGTTGCCATCATT | Qadis et al., 2014 |
| Enterococcus-R | ACTCGGTTGTACTTCCCATTGT |  |
| Pseudomonas-F | GACGGGTGAGTAATGCCTA | Roosa et al., 2014 |
| Pseudomonas-R | CACTGGTGTTCCTTCCTATA |  |
| Methylobacterium-F | GGTGTTCTTGCGAATATCT | Bal et al., 2013 |
| Methylobacterium-R | GGACGCTTGAGTATGGTA |  |
| Cyanobacteria-F | GGGGAATYTTCCGCAATGGG | Nübel et al., 1997 |
| Cyanobacteria-R | GACTACTGGGGTATCTAATCCCATT |  |
| Ralstonia-F | AGAGGTCGACGCGATACAGT | Singh et al., 2014 |
| Ralstonia-R | CATGAGCAAGGACGAAGTCA |  |
| Metabolic genes | | |
| endoglucanase-F | AAATGGCTGAGGGACGATTGGG | This study |
| endoglucanase-R | TGCCGCTTCAACCGCTTCTT |  |
| endo-1,4-beta-xylanase-F | GTCAATGGGTCTGGCGGGAATT | This study |
| endo-1,4-beta-xylanase-R | GCGATCTCGTCCAGCCATACAA |  |
| mgsA-F | ATGGAACTGACGACTCGCAC | Zhao et al., 2019 |
| mgsA-R | TTACTTCAGACGGTCCGCGAG |  |
| subtilisin-F | CCATTGCGGTAGGTGCGGTAA | This study |
| subtilisin-R | CGTAAGTGCCTCCAGGAAGTGT |  |
| IL-4-F | CTCCCAACTGATCCCAACCC | Zhang et al., 2018 |
| IL-4-R | TGCACGAGTTCTTTCTCGCT |  |
| IL-6-F | AGGGAAATGTCGAGGCTGTG | Zhang et al., 2018 |
| IL-6-R | TCCACTCGTTCTGTGACTGC |  |
| β-actin-F | TGAGCTGCGTTTTACACCCT | Zong et al., 2019 |
| β-actin-R | GCCTTCACCGTTCCAGTTTTT |  |

**Supplementary References**

Bal Krishna, K C, Sathasivan A, Ginige MP. 2013. Microbial community changes with decaying chloramine residuals in a lab-scale system. Water Res 47(13):4666-79.

Nübel U, Garcia-Pichel F, Muyzer G. 1997. PCR primers to amplify 16S rRNA genes from *Cyanobacteria*. Appl Environ Microbiol 63(8):3327-32.

Qadis AQ, Goya S, Ikuta K, Yatsu M, Kimura A, Nakanishi S, Sato S. 2014. Effects of a bacteria-based probiotic on ruminal pH, volatile fatty acids and bacterial flora of Holstein calves. J Vet Med Sci 76(6):877–885.

Roosa S, Wauven CV, Billon G, Matthijs S, Wattiez R, Gillan DC. 2014. The *Pseudomonas* community in metal-contaminated sediments as revealed by quantitative PCR: a link with metal bioavailability. Res Microbiol 165(8):647-56.

Singh D, Sinha S, Yadav DK, Chaudhary G. 2014. Detection of *Ralstonia solanacearum* from asymptomatic tomato plants, irrigation water, and soil through non-selective enrichment medium with hrp gene-based bio-PCR. Curr Microbiol 69(2):127-34.

Zhang Y, Shi CY, Wang C, Lu ZQ, Wang FQ, Feng J, Wang YZ. 2018. Effect of soybean meal fermented with *Bacillus subtilis* BS12 on growth performance and small intestinal immune status of piglets. Food Agric Immunol 29(1):133-146.

Zhao C, Dong H, Zhang Y, Li Y. 2019. Discovery of potential genes contributing to the biosynthesis of short-chain fatty acids and lactate in gut microbiota from systematic investigation in *E. coli*. NPJ Biofilms Microbiomes 5:19.

Zong X, Cao XX, Wang H, Xiao X, Wang YZ, Lu ZQ. 2019. Cathelicidin-WA facilitated intestinal fatty acid absorption through enhancing PPAR-gamma dependent barrier function. Front Immunol 10:1674.
