# Supplementary material for: Dynamics of the Physicochemical Characteristics, Microbiota, and Metabolic Functions of Soybean Meal and Corn Mixed Substrates during Two-Stage Solid-State Fermentation
Source: mSystems. 2020 Feb 11;5(1):e00501-19. doi: 10.1128/mSystems.00501-19 (PMC7018524; doi:10.1128/mSystems.00501-19)
Supplement: TABLE S3 [file mSystems.00501-19-st003.docx]

**Table S3.**

| Item | Control | 10% FMS |
| --- | --- | --- |
| Ingredients, % |  |  |
| Corn | 53.90 | 50.10 |
| Extruded soybean | 10.00 | 10.00 |
| Soybean meal, dehulled | 21.10 | 15.90 |
| FMS | - | 10.00 |
| Wheat bran | 2.00 | 1.00 |
| Low protein whey powder | 3.00 | 3.00 |
| Fish meal | 3.00 | 3.00 |
| Soy oil | 2.00 | 2.00 |
| Glucose | 2.00 | 2.00 |
| Calcium hydrophosphate | 0.75 | 0.70 |
| Limestone | 0.60 | 0.65 |
| Lysine | 0.45 | 0.45 |
| Methionine | 0.12 | 0.12 |
| Threonine | 0.15 | 0.15 |
| Salt | 0.50 | 0.50 |
| Premix | 0.43 | 0.43 |
| Total | 100.0 | 100.0 |
| Analyzed composition |  |  |
| Dry matter | 88.85±0.24 | 87.59±0.16 |
| Crude protein | 19.80±0.38 | 19.79±0.29 |
| Ether extract | 8.33±0.18 | 8.33±0.21 |
| Calcium | 0.93±0.03 | 1.07±0.04 |
| Phosphorus | 0.49±0.09 | 0.65±0.13 |
